# Development of an adult whole-body PBPK model of irinotecan and its metabolites for predicting UGT1A1/CYP3A-mediated drug-drug interactions

**DOI:** 10.3389/fphar.2026.1801897

**Published:** 2026-05-26

**Authors:** Bowen Chen, Longjie Li, Wenhang Xu, Haiping Xu, Xinyan Zhu, Qingfeng He, Xiao Zhu, Paul Chi-Lui Ho, Xiaoqiang Xiang, Peiying Ji

**Affiliations:** 1 Department of Clinical Pharmacy and Pharmacy Administration, School of Pharmaceutical Sciences, Fudan University, Shanghai, China; 2 School of Pharmacy, Monash University Malaysia, Subang Jaya, Malaysia; 3 Quzhou Fudan Institute, Quzhou, China; 4 State Key Laboratory of Advanced Drug Formulations for Overcoming Delivery Barriers, Shanghai, China; 5 Department of Pharmacy, Kong Jiang Hospital of Yangpu District, Shanghai, China

**Keywords:** cyp3a, drug-drug interactions, irinotecan, physiologically based pharmacokinetic model, SN-38, UGT1A1

## Abstract

**Background:**

Irinotecan (CPT-11) is a prodrug that is bioactivated to SN-38, which is primarily cleared via UGT1A1-mediated glucuronidation. Its complex metabolism, which also involves CYP3A-mediated oxidation, makes irinotecan highly susceptible to enzyme-mediated drug-drug interactions (DDIs). This study aimed to develop and qualify an adult whole-body physiologically based pharmacokinetic (PBPK) model of irinotecan and its metabolites (SN-38, SN-38G, and APC) to predict systemic pharmacokinetics and UGT1A1/CYP3A-mediated DDIs.

**Methods:**

An adult middle-out PBPK model was implemented in Simcyp®, integrating key pathways for carboxylesterase (CES) -mediated activation, UGT1A1/UGT1A9-driven glucuronidation, and CYP3A4/5-mediated oxidation. The model was calibrated using 175–300 mg/m^2^ monotherapy data and was validated across a 33–750 mg/m^2^ dose range, including drug-drug interactions with ketoconazole, sorafenib, and lopinavir/ritonavir.

**Results:**

Most observed irinotecan and SN-38 concentrations fell within the fifth–95th percentile prediction intervals, and dose–exposure slopes were close to unity. Across monotherapy evaluations, 54/56 (96.4%) comparisons of maximum concentration (C_max_) and area under the concentration–time curve (AUC) met the 2-fold acceptance criterion. The model further captured the direction and magnitude of exposure changes across all inhibitor scenarios, with CPT-11 and SN-38 exposure ratios remaining within acceptable limits, although APC exposure was overpredicted in the ketoconazole scenario.

**Conclusion:**

The mechanistically informed and validated whole-body PBPK model reliably describes dose-dependent irinotecan and SN-38 pharmacokinetics and UGT1A1/CYP3A-mediated drug-drug interactions. This framework provides a clinically relevant tool for enzyme-mediated DDI risk assessment and for exploratory simulation of dose–exposure relationships under standard irinotecan regimens.

## 1 Introduction

**FIGURE 1:**
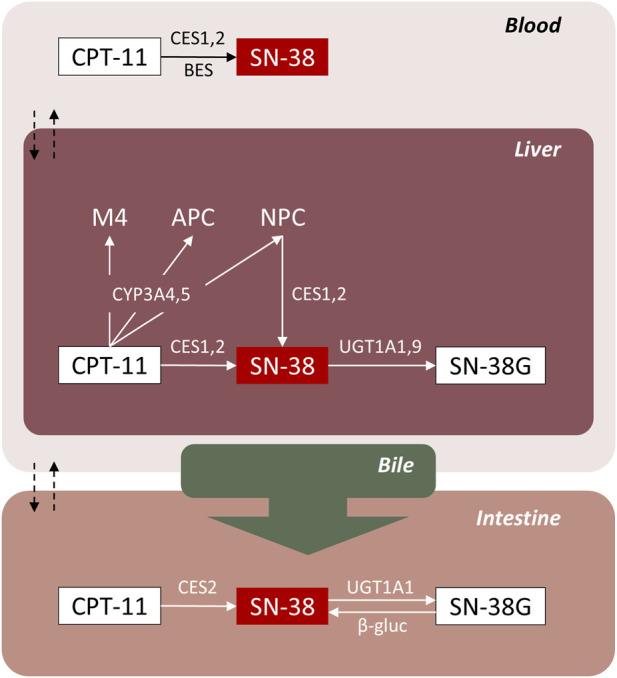
Simulated tissue distribution and mechanistic disposition mechanism of irinotecan, SN-38, and SN-38G. CPT-11: Irinotecan; CES1,2: carboxylesterase-1 and carboxylesterase-2; SN-38: 7-ethyl-10-hydroxycamptothecin; SN-38G: SN-38 glucuronide; CYP: Cytochrome P450; UGT: Uridine diphosphate–glucuronosyltransferase; OATP1B1: Organic anion transporting polypeptide 1B1; P-gp: P-glycoprotein; MRP2: Multidrug resistance-associated protein 2; BCRP: Breast cancer resistance protein.

Irinotecan (CPT-11) is a first-line chemotherapeutic agent for colorectal and pancreatic cancers. It is a prodrug that requires metabolic activation to SN-38, the topoisomerase inhibitor responsible for both antitumor efficacy and gastrointestinal (GI) toxicity (Mathijssen et al., 2001; Sun et al., 2020). The disposition of CPT-11 is governed by a multi-enzyme, multi-compartment network. Conversion to SN-38 is mediated primarily by carboxylesterase-2 (CES2), with a minor contribution from CES1. In contrast, SN-38 is eliminated mainly through UGT1A1-catalyzed glucuronidation to SN-38G (Ma and McLeod, 2003). In parallel, CPT-11 undergoes CYP3A4/5-mediated oxidation to APC, which constitutes a major competing clearance pathway (Rivory, 2000; Sai et al., 2001). This oxidative route yields additional minor metabolites, including M4 and NPC. Enterohepatic cycling (EHC) further redistributes CPT-11, SN-38, and SN-38G to the gut lumen, where microbial β-glucuronidase (GUS) can regenerate SN-38 from SN-38G and contribute to delayed intestinal toxicity (Takasuna et al., 1996; Wallace et al., 2010). Transporters such as P-glycoprotein (P-gp), breast cancer resistance protein (BCRP), and OATP family transporters (OATP) have been implicated in systemic and tissue disposition (Mathijssen et al., 2002), although their quantitative in-vivo contributions remain insufficiently defined. Collectively, these processes form a tightly coupled network in which modest perturbations can propagate nonlinearly to alter SN-38 exposure. Figure 1 summarizes the tissue distribution and mechanistic disposition pathways of irinotecan, SN-38, and SN-38G. Recent quantitative studies integrating in-vitro kinetics, tissue proteomics, and microbiome activation data have begun to define organ-specific determinants of intestinal SN-38 burden and interindividual variability (Parvez et al., 2021).

Owing to this complex metabolic architecture, irinotecan serves as an informative model compound for studying metabolite-mediated drug-drug interactions (DDIs). Clinically relevant perpetrator drugs target specific nodes within this network: ketoconazole, a potent CYP3A inhibitor, suppresses APC formation, thereby shunting metabolic flux toward CES-mediated SN-38 generation. Sora+nib primarily inhibits UGT1A1 (with ancillary CYP3A inhibition), leading to a preferential accumulation of SN-38 relative to CPT-11. In contrast, the lopinavir/ritonavir combination concurrently inhibits both UGT1A1 (via lopinavir) and CYP3A (via ritonavir), resulting in a pronounced SN-38-dominant interaction profile (Kehrer et al., 2002; Mross et al., 2007; Corona et al., 2008). Consequently, the net change in drug exposure reflects the dynamic balance between metabolic activation (CES), oxidative clearance (CYP3A), and conjugative elimination (UGT1A1), rather than an isolated effect on any single pathway.

However, these mechanistic perturbations are superimposed on substantial design- and population-dependent variability. Design features such as dose matching, infusion duration, perpetrator loading strategy, and whether CPT-11 is administered at perpetrator steady state can materially influence the apparent DDI magnitude. For instance, a pivotal ketoconazole DDI study used non-dose-matched comparators (350 mg/m^2^ monotherapy vs. 100 mg/m^2^ with ketoconazole), which obscures a straightforward interpretation of the exposure ratios (Kehrer et al., 2002). Adding another layer of complexity, population-level heterogeneity in the form of ethnicity-dependent differences in UGT1A1 and CES expression and activity—evidenced by varying frequencies of UGT1A1*28, UGT1A1*6, and dual-variant genotypes across Caucasian, East Asian, and admixed populations—alters the baseline clearance of SN-38, thereby modulating the observed DDI magnitude even under nominally identical dosing conditions (Minami et al., 2007; Xu et al., 2002).

Physiologically based pharmacokinetic (PBPK) modeling offers a mechanistic framework to integrate physiological, biochemical, and drug-specific data, holding great promise for predicting complex drug interactions (Taskar et al., 2025). However, these intertwined clinical, mechanistic, and demographic challenges have historically hampered the development of predictive PBPK models for irinotecan. Prior PBPK efforts have typically focused on limited portions of the network—such as single-pathway descriptions of SN-38 formation or partial representations of CYP3A-mediated oxidation and EHC—and often treated enterohepatic cycling empirically, with transporter involvement either omitted or included using weakly constrained kinetic priors (Fan et al., 2017; Fan et al., 2019). Such simplifications can obscure key flux-redistribution (metabolic shunting) effects, particularly under concomitant inhibition of multiple pathways. Consequently, the prospective prediction of irinotecan DDIs and the reconciliation of seemingly conflicting clinical data have remained a significant challenge.

To address these limitations, we developed and qualified an adult middle-out whole-body PBPK model describing CPT-11 and its metabolites SN-38, SN-38G, and APC. The model is mechanistically transparent and specifically designed for DDI risk assessment. It incorporates CES1/2-mediated activation, UGT1A1-driven glucuronidation (with minor UGT1A9 contribution), and CYP3A4/5-mediated APC formation, while representing enterohepatic cycling using an effective-clearance approach that preserves mass balance without overparameterizing biliary or intestinal processes. Transporters were not modeled as explicit mechanistic modules, not because transporter effects were assumed absent, but because currently available clinical datasets do not provide sufficient transporter-selective or tissue-level constraints to identify uptake and efflux processes in parallel with CES-, UGT-, and CYP-mediated pathways. Accordingly, the present framework was developed as an enzyme-focused whole-body PBPK model for UGT1A1/CYP3A-mediated DDI assessment. Following calibration with monotherapy data, the model was prospectively qualified using clinical DDI studies with ketoconazole, sorafenib, and lopinavir/ritonavir. Within this intended scope, the model supports enzyme-mediated interaction risk assessment and exploratory dose optimization under standard irinotecan regimens.

## 2 Methods

### 2.1 Software and data

All PBPK simulations were performed using Simcyp® (Version 24), employing the default adult full–PBPK model structure and built-in compound and population libraries. Digitization of graphical pharmacokinetic data was carried out using WebPlotDigitizer (Version 4.6).

Clinical input data for model development and qualification were derived exclusively from published human studies. Intravenous irinotecan monotherapy regimens (33–750 mg/m^2^; 90-min infusions) and the clinical DDI study designs involving ketoconazole, sorafenib, and lopinavir/ritonavir were implemented according to the original trial protocols (Kehrer et al., 2002; Mross et al., 2007; Corona et al., 2008; de Jonge et al., 2000; Abigerges et al., 1995; Catimel et al., 1995). Plasma concentration–time profiles available only in graphical form were digitized using WebPlotDigitizer, whereas tabulated values for C_max_, AUC, or exposure ratios were extracted directly when reported. No new clinical or bioanalytical measurements were performed for this study; all monotherapy and DDI pharmacokinetic data were obtained from published trials.

Compound-specific physicochemical, binding, and enzyme-kinetic parameters were collated from primary literature, public databases, Simcyp® predictions, and, when required, in-silico tools. For CPT-11 and SN-38, experimentally measured or curated database values (e.g., DrugBank) were used whenever available and served as the primary sources for physicochemical and blood-binding parameters. For SN-38G and especially APC, experimentally determined properties were limited; missing descriptors—including logP, pKa, plasma unbound fraction (fu,p), and blood-to-plasma ratio (B:P)—were therefore initialized using ADMET Predictor®. Tissue-to-plasma partition coefficients (K_p_) for all analytes were predicted using the Rodgers–Rowland method within Simcyp® (Rodgers and Rowland, 2007; Abigerges et al., 1995). ADMET-derived properties and the global K_p_ scalar were treated as prior values and were allowed to vary only within physiologically plausible bounds during calibration to recover observed steady-state volume of distribution (V_SS_) and overall systemic exposure. Final parameter values and their sources are summarized in Table 3.

### 2.2 Irinotecan PBPK model development

A whole-body physiologically based pharmacokinetic (PBPK) model for irinotecan and its metabolites was implemented in Simcyp® (Version 24), using the default adult full-PBPK structure and physiological parameters. The disposition network incorporated three primary biochemical pathways.

1. Carboxylesterase-mediated activation, converting irinotecan to SN-38 predominantly via CES2 with minor CES1 contribution;
2. UGT1A1-and UGT1A9-mediated glucuronidation, forming SN-38G; and
3. CYP3A4/5-mediated oxidation, producing the metabolite APC.

Each metabolic pathway was parameterized using literature-reported kinetic constants (V_max_, K_m_) and enzyme-abundance values from human liver microsomes and recombinant enzyme systems (Ma and McLeod, 2003; Rivory, 2000; Sai et al., 2001; Parvez et al., 2021; Xiao et al., 2018).

To capture enzyme kinetics quantitatively, microsomal and recombinant kinetic parameters (V_max_ and K_m_) for CES1, CES2, UGT1A1, UGT1A9, and CYP3A4/5 were compiled from studies using human liver microsomes or recombinant enzyme preparations. Intrinsic clearances were subsequently scaled to whole-organ values using established physiological scalars, including microsomal protein per gram of liver (MPPGL), hepatocellularity factors, and enzyme abundance measurements in hepatic and intestinal tissues. When multiple literature sources were available for a given parameter, central estimates were selected as the initial values, and uncertainty ranges were defined based on the reported variability, ensuring physiologically supported parameter bounds.

Biliary excretion of irinotecan, SN-38, and SN-38G was incorporated using literature-based intrinsic clearances. EHC was represented through an empirical intrinsic biliary clearance term transporting drug from the liver to the gut lumen. This implementation preserved systemic mass balance and the overall impact of EHC on CPT-11/SN-38 pharmacokinetics without explicitly modeling gallbladder emptying, intestinal β-glucuronidase activity, or segment-specific reabsorption.

Although membrane transporters—including P-gp, BCRP, and OATP1B1, OATP1B3, OATP2B1—have been implicated in the disposition of irinotecan and SN-38, quantitative in-vivo kinetic data remain sparse and heterogeneous. Incorporating multiple transporter modules alongside the CES-, UGT-, and CYP-mediated pathways would lead to parameter non-identifiability and structural over-parameterization. Therefore, transporter contributions were handled implicitly, absorbed into organ-specific intrinsic clearance terms rather than modeled as separate mechanistic pathways Although membrane transporters—including P-gp, BCRP, and OATP1B1/1B3/2B1—have been implicated in the disposition of irinotecan and SN-38 (Mathijssen et al., 2002), transporter effects were not assumed absent in the present work. Rather, explicit transporter modules were intentionally omitted because transporter-specific in-vivo kinetic priors and transporter-selective clinical constraints remain sparse, heterogeneous, and insufficient to uniquely identify multiple uptake and efflux processes in parallel with CES-, UGT-, and CYP-mediated pathways using the plasma PK endpoints available here. Under these data conditions, adding explicit transporter terms would be more likely to increase structural flexibility and parameter non-identifiability than to improve mechanistic certainty. Therefore, transporter contributions were handled implicitly within organ-specific clearance terms, and the model should be interpreted as an enzyme-focused rather than transporter-inclusive PBPK framework.

During model calibration, only a restricted subset of parameters—including CES-, UGT1A1/UGT1A9-, and CYP3A4/5-mediated metabolic clearances, biliary intrinsic clearance (CLint, bile), and the global Kp scalar—was allowed to vary. All adjustments were constrained within approximately two-fold of literature-based or in-silico priors, ensuring physiologic plausibility and preventing model overfitting.

### 2.3 Perpetrator drug models development

Perpetrator drugs were implemented using Simcyp® compound templates and published PBPK model profiles to reproduce their pharmacokinetic and inhibitory characteristics under the clinical DDI regimens. Ketoconazole was modeled as a strong reversible and time-dependent inhibitor of CYP3A. Baseline pharmacokinetic parameters, including oral bioavailability, clearance, and half-life, were initialized from the Simcyp® ketoconazole template and Ki was refined within literature-reported parameter ranges to match observed exposures reported in the irinotecan–ketoconazole study (Kehrer et al., 2002). Sorafenib was implemented as an oral multikinase inhibitor with inhibitory components affecting both UGT1A1 and CYP3A. Absorption and clearance parameters were initialized from published single-agent pharmacokinetic profiles, then modestly adjusted Ki to recover observed plasma concentrations at the clinical dose of 400 mg twice daily in the DDI study (Mross et al., 2007). For lopinavir/ritonavir, linked compound files were used to represent the combined regimen, with ritonavir acting as a potent inhibitor and modulator of CYP3A-mediated clearance, while lopinavir contributed inhibitory effects on UGT1A1. Pharmacokinetic profiles for the lopinavir/ritonavir regimen were first reproduced under single-agent conditions, after which irinotecan was added to simulate the reported two-period within-cycle study design (Corona et al., 2008). For each perpetrator drug, a single global parameter set was established and subsequently applied across all irinotecan DDI simulations involving that perpetrator. No study-specific retuning was performed once the perpetrator model had been finalized. Final parameter values, optimization bounds, and corresponding literature sources are summarized in Supplementary Tables S1–S5.

### 2.4 Design of virtual regimens and model evaluation

Simulations were performed using the Simcyp® Healthy Volunteer population (ages 20–50 years, 50% female), with default interindividual variability applied to physiology, enzyme expression, and plasma protein binding. Unless specified otherwise, each scenario consisted of 10 virtual trials of 10 subjects (n = 100). Three categories of irinotecan monotherapy designs reported in clinical studies were reproduced: therapeutic-dose regimens (175–300 mg/m^2^) for primary calibration (de Jonge et al., 2000); (ii) high-dose regimens (100–750 mg/m^2^) to assess dose proportionality and model scalability (Abigerges et al., 1995); (iii) and low-dose or repeated-dose schedules (33–115 mg/m^2^) to evaluate UGT-mediated clearance at lower exposures (Catimel et al., 1995).

Drug–drug interaction simulations followed each clinical study design. The ketoconazole interaction was reproduced using a two-cycle, non-dose-matched design (350 mg/m^2^ monotherapy vs. 100 mg/m^2^ with ketoconazole) (Kehrer et al., 2002). For sorafenib, subjects received 400 mg twice daily to steady state before irinotecan dosing with sampling to 48 h (Mross et al., 2007). For lopinavir/ritonavir, a within-cycle two-period design was implemented, withholding LPV/RTV before monotherapy dosing and coadministering the morning LPV/RTV dose at the start of infusion during the combination period (Corona et al., 2008).

**FIGURE 2:**
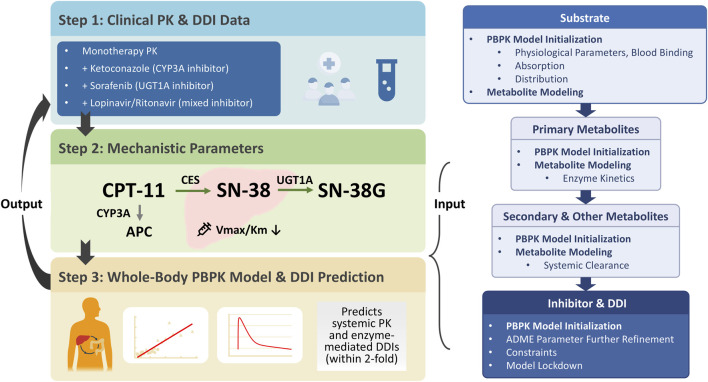
Structured workflow of the DDI PBPK model development for irinotecan and its metabolites.

Clinical datasets were prospectively allocated across distinct stages of model development, as summarized in Table 1. Model development was conducted within a predefined staged framework in which monotherapy datasets established baseline disposition and metabolite formation parameters, and selected DDI studies informed further refinement of predefined pharmacokinetic parameters. Once a single global parameter set demonstrating acceptable performance across calibration datasets was obtained, it was finalized and locked. External validation and qualification were then conducted using datasets reserved *a priori* for this purpose, including monotherapy and interaction datasets as summarized in Table 1. Model performance was evaluated by comparing observed and predicted Cmax, AUC, and exposure ratios, supported by fold-error analyses using a 2-fold acceptance boundary (Shebley et al., 2018). The overall structural and sequential workflow of model construction is summarized in Figure 2.

**TABLE 1:** Allocation of clinical datasets across model development stages.

Dataset/Study	Dose/Design	Application stage
de Jonge et al.	Monotherapy, 175–300 mg/m^2^	Model development
Abigerges et al.	Monotherapy, 100–750 mg/m^2^	Qualification
Catimel et al.	Monotherapy, 33–115 mg/m^2^	Qualification
Kehrer et al. (ketoconazole)	DDI, CYP3A	Model development
Mross et al. (sorafenib)	DDI, CYP3A+UGT1A	Model development
Corona et al. (lopinavir/ritonavir)	DDI, CYP3A+UGT1A	Qualification

## 3 Results

### 3.1 Predictive performance for irinotecan PBPK model

**FIGURE 3:**
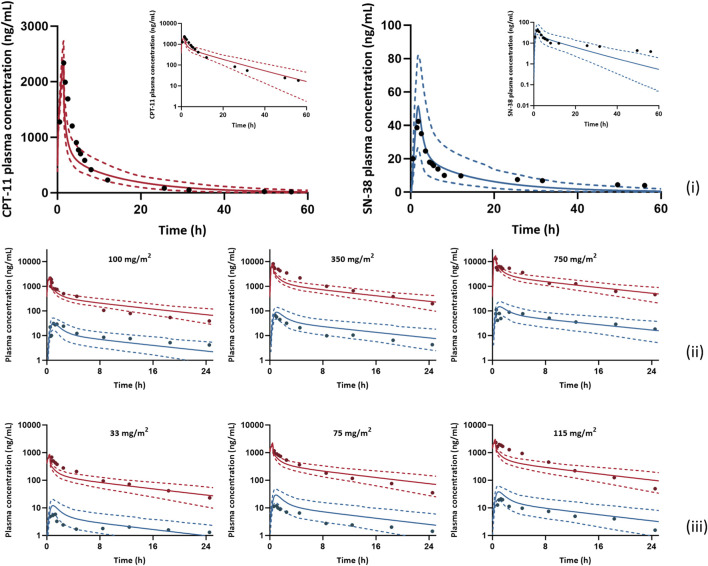
Observed and PBPK-predicted CPT-11 (red) and SN-38 (blue) plasma profiles following 90-min IV monotherapy across three clinical studies. Panels (i)–(iii) correspond to 200 mg/m^2^; 100/350/750 mg/m^2^; and 33/75/115 mg/m^2^, respectively. Solid lines: median; dashed lines: fifth-95th percentiles; symbols: observations.

The PBPK model adequately reproduced the plasma concentration–time profiles of CPT-11 and SN-38 across three monotherapy datasets spanning 33–750 mg/m^2^. Predicted medians and fifth–95th percentile intervals encompassed the observed variability in all studies, including intermediate single-dose regimens (de Jonge et al., 2000), every-3-week dosing at 100–750 mg/m^2^ (Abigerges et al., 1995), and low-dose repeated daily schedules (Catimel et al., 1995). Across this dose range, the model captured both the rapid distribution phase and the terminal elimination phase without consistent underprediction or overprediction of concentrations (Figure 3). The study-specific offsets observed for AUC likely reflect differences in trial design (e.g., dosing schedule and sampling duration) and population heterogeneity, including interindividual variability in CES/UGT1A1 expression and genotype distributions across studies. Therefore, model evaluation emphasized within-study agreement and overall dose–exposure trends, rather than requiring identical absolute AUC levels across heterogeneous trials.

Consistent with the visual agreement, predicted C_max_ and AUC values showed strong concordance with observed data (Supplementary Table S6). Under a 2-fold acceptance criterion, 54 of 56 comparisons (96.4%) for CPT-11 and SN-38 C_max_ and AUC fell within the acceptable range. At therapeutic doses (175–300 mg/m^2^), Fold error (FE) for CPT-11 were 0.87–1.18 for C_max_ and 0.92–1.28 for AUC, while FE values for SN-38 ranged from 1.42–1.66 for C_max_ and 0.67–1.81 for AUC.

**FIGURE 4:**
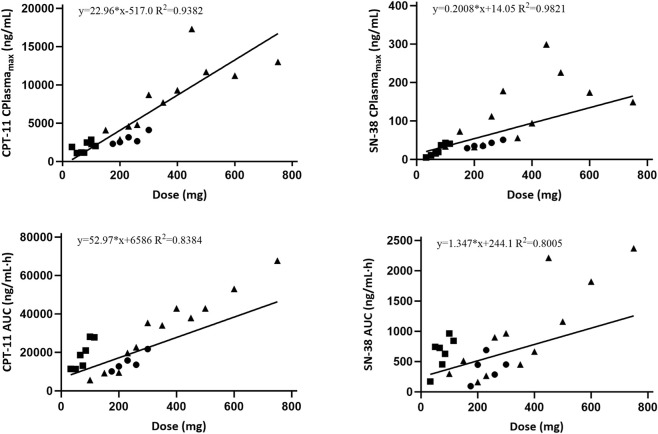
Dose–exposure relationships for CPT-11 and SN-38 under monotherapy (90-min IV infusion). Panels show C_max_ (top) and AUC (bottom) versus irinotecan dose across three clinical studies. Different symbols denote different studies: Data points correspond to three clinical studies: circles indicate de Jonge et al. (2000), triangles indicate Abigerges et al. (1995), and squares indicate Catimel et al. (1995). Solid lines represent linear regression fits.

Across the three monotherapy trials, C_max_ and AUC for CPT-11 and SN-38 increased approximately linearly over the 33–750 mg/m^2^ range (Figure 4). Dose–exposure regressions yielded slopes close to unity, with no systematic deviation at either the lower or upper dose levels, supporting the dose scalability of the model within the investigated range.

### 3.2 Predictive performance for drug-drug interactions

**FIGURE 5:**
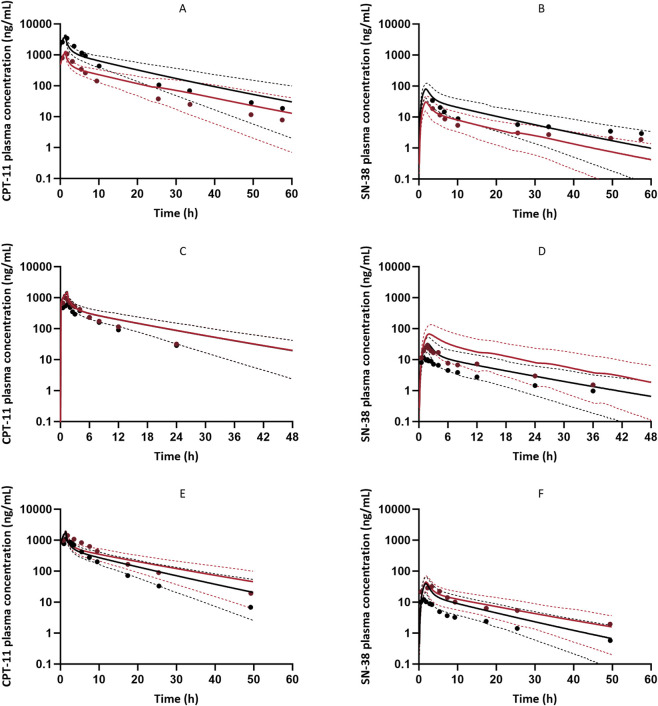
Observed and PBPK-predicted concentration–time profiles under DDIs **(A,B)** Ketoconazole **(C,D)** sorafenib **(E,F)** lopinavir/ritonavir. Solid lines denote median predictions; dashed lines denote the fifth–95th percentiles; symbols are observed data. Note: the ketoconazole study used non–dose-matched comparators (350 mg/m^2^ monotherapy vs. 100 mg/m^2^ with ketoconazole); curves are shown for qualitative comparison. Black: monotherapy; red: DDI.

DDI evaluations showed that the model accurately recovered both the direction and magnitude of exposure changes for ketoconazole, sorafenib, and lopinavir/ritonavir under their respective clinical designs (Figure 5; Table 2). For CPT-11 and SN-38, all predicted DDI/control ratios for Cmax and AUC were within 2-fold of the observed values. Ratios for SN-38G and APC were generally acceptable, with the only notable exception being APC AUC in the ketoconazole study.

**TABLE 2:** Predicted vs. observed exposure ratios (DDI/control) for irinotecan and its metabolites (C_max_ and AUC).

Analyte	Prediction		Observation		FE_Cmax, ratio_	FE_AUC, ratio_
​	C_max_ ratio	AUC ratio	C_max_ ratio	AUC ratio		
Ketoconazole						
CPT-11	0.298	0.347	0.278	0.258	1.072	1.344
SN-38	0.383	0.392	0.537	0.538	0.713	0.728
APC	0.049	0.114	0.064	0.030	0.763	3.811
SN-38G	0.388	0.395	0.442	0.541	0.877	0.731
SOR						
CPT-11	1.037	1.199	1.360	1.260	0.762	0.952
SN-38	2.096	2.648	2.220	2.200	0.944	1.203
LPV/RTV						
CPT-11	1.042	1.334	1.145	1.861	0.910	0.717
SN-38	1.797	2.521	2.340	2.861	0.768	0.881
APC	0.027	0.039	0.162	0.153	0.167	0.255
SN-38G	1.175	1.467	1.394	1.877	0.842	0.781

FE_Cmax, ratio_ = C_max, predicted_/C_max, observed_; FE_AUC, ratio_ = AUC_predicted_/AUC_observed_.

Ratios are defined as DDI/control (coadministration/monotherapy).

In the ketoconazole study, the non–dose-matched design resulted in lower CPT-11 and SN-38 concentrations during coadministration; these profiles are therefore interpreted qualitatively (Figures 5A,B). Nonetheless, model-predicted DDI/control ratios for CPT-11 and SN-38 showed good concordance with observations, with all fold errors remaining within the standard two-fold acceptance range. APC exposure was overpredicted, whereas the SN-38G ratio remained acceptable. Summary metrics are presented in Table 2.

In the dose-matched sorafenib study, the model reproduced the observed increases in CPT-11 and SN-38 exposure (Figures 5C,D), and all predicted DDI/control ratios fell within the two-fold boundary. Corresponding ratio data are summarized in Table 2.

With lopinavir/ritonavir, the model captured the expected modest increases in CPT-11 and the larger increases in SN-38 (Figures 5E,F). Predicted ratios closely matched observations, and the fold errors for CPT-11, SN-38, APC, and SN-38G all remained within two-fold. Full results are listed in Table 2.

Overall, the monotherapy and DDI results demonstrate strong predictive performance for systemic CPT-11 and SN-38 exposure across a broad dose range and three mechanistically distinct perpetrators. In total, 96.4% of monotherapy Cmax/AUC comparisons and all CPT-11/SN-38 DDI ratios met the 2-fold acceptance standard, with APC AUC under ketoconazole as the single consistent outlier. These findings support the suitability of the model for enzyme-mediated interaction risk assessment and exploratory dose-adjustment simulations in conventional irinotecan treatment regimens.

## 4 Discussion

The present work developed and qualified an adult whole-body, middle-out PBPK model for CPT-11, SN-38, SN-38G, and APC that integrates CES-mediated activation, UGT1A1-driven glucuronidation, and CYP3A-mediated oxidation within a unified disposition network. The model successfully reproduced CPT-11 and SN-38 pharmacokinetics across three independent monotherapy datasets over the 33–750 mg/m^2^ dose range after 90-min intravenous infusions, capturing the approximately linear dose–exposure relationship. Under a 2-fold evaluation criterion, monotherapy and DDI simulations showed generally good agreement with observed C_max_ and AUC, while highlighting a residual discrepancy for APC AUC under ketoconazole. Taken together, these results support the use of the model for enzyme-mediated interaction risk assessment and exploratory dose-adjustment simulations in standard irinotecan regimens.

Several physiologically based pharmacokinetic (PBPK) models of irinotecan have previously been reported. For example, Fan et al. (2017) developed a whole-body PBPK model describing the disposition of irinotecan and its major metabolites (SN-38, SN-38G, and APC), primarily for systemic exposure prediction and mechanistic understanding of metabolic pathways. Toshimoto et al. (2017) further extended a WB-PBPK framework to perform virtual clinical studies, focusing on the probability distribution of AUC at target tissues and evaluating the impact of genetic polymorphisms of metabolic enzymes and transporters on irinotecan-induced side effects. More recently, Tao et al. (2022) developed a PBPK model incorporating inflammation-mediated alterations in metabolic enzymes and transporter expression, with emphasis on mechanistic investigation and cross-species extrapolation. While these studies have substantially advanced mechanistic understanding of irinotecan pharmacokinetics, most were not systematically qualified for quantitative drug–drug interaction (DDI) prediction across multiple inhibitors and a broad clinical dose range. In contrast, the present model was specifically developed and qualified for enzyme-mediated DDI assessment focused on UGT1A1/CYP3A perturbation. It was calibrated using 175–300 mg/m^2^ monotherapy data and externally validated across a wide dose range (33–750 mg/m^2^). Furthermore, the model captured exposure changes under CYP3A- and UGT1A1-focused inhibitor scenarios (ketoconazole, sorafenib, and lopinavir/ritonavir), with more than 95% of Cmax and AUC predictions within the two-fold acceptance criterion. Therefore, while building upon previously published mechanistic PBPK frameworks, the current study adds value by providing a systematically qualified, enzyme-focused PBPK platform for DDI risk assessment and exploratory dose optimization under standard irinotecan regimens.

The three perpetrator regimens were used here as clinically relevant enzyme-focused interaction scenarios. Although transporter modulation by these perpetrators cannot be excluded, the observed clinical DDIs were interpreted within the present framework primarily through CYP3A- and/or UGT1A1-mediated perturbation. Ketoconazole acts primarily as a strong CYP3A inhibitor. In the current model, inhibition of the CPT-11→APC oxidative branch decreases this clearance pathway and proportionally increases the fraction of CPT-11 available for CES-mediated activation. Under dose-matched conditions, such metabolic shunting would be expected to increase both CPT-11 and SN-38 exposure without invoking esterase induction. Although the reference ketoconazole trial used non–dose-matched comparators (350 mg/m^2^ monotherapy vs. 100 mg/m^2^ with ketoconazole), complicating naïve evaluation of concentration–time profiles, the predicted DDI/control ratios for CPT-11 and SN-38 nevertheless aligned closely with observations (Table 3). Sorafenib exerts a primary inhibitory effect on UGT1A1 with a secondary contribution from CYP3A (Mross et al., 2007). In the dose-matched combination study (125 mg/m^2^ irinotecan), this produced modest increases in CPT-11 exposure and more pronounced increases in SN-38, consistent with reduced conjugative clearance from SN-38 to SN-38G. The model reproduced these signatures, supporting the representation of UGT1A1 as the dominant SN-38 clearance pathway with UGT1A9 playing a smaller role (Parvez et al., 2021; Xiao et al., 2018).

**TABLE 3:** PBPK input parameters for irinotecan (CPT-11) and its metabolites SN-38, SN-38G and APC.

Category	Parameter	Value	Source
Irinotecan (CPT-11)			
Physicochemical and blood binding	MW (g/mol)	586.678	Drugbank
	log $P_{o:w}$	2.78	
	Compound type	Ampholyte	
	p $K_{a}$ 1	11.71	
	p $K_{a}$ 2	9.47	
	B/P ratio	1.2	Simcyp® prediction
	${\text{fu}}_{p}$	0.30	Chabot (1997)
Absorption (ADAM model)	$P_{\text{eff},\text{man}}$ ( $10^{-4}$ cm/s)	0.87	Simcyp® prediction
	Polar surface area, PSA (Å^2^)	112.51	DrugBank
	Hydrogen bond donors (HBD)	1	
Distribution (full PBPK model)	Volume of distribution at steady state, $V_{\text{SS}}$ (L/kg)	4.987	Simcyp® prediction
	$K_{p}$ scalar	0.8	Chabot (1997); Xie et al. (2002), Atasilp et al. (2018)
Elimination – enzyme kinetics	CYPs (recombinant)		
	CYP3A4 ${\text{CL}}_{\mathrm{int}}$ (µL/min/pmol)	0.09	de Man et al. (2018)
	Carboxylesterases (microsomal)		
	CES1 $V_{\max}$ (pmol/min/pmol isoform)	6.36	Optimized ( Humerickhouse et al., 2000; Slatter et al., 1997; Parvez et al., 2021; Satoh et al., 1994; Rivory et al., 1996 )
	CES1 $K_{M}$ (µM)	42.7	Humerickhouse et al. (2000)
	CES2 $V_{\max}$ (pmol/min/pmol isoform)	30	Optimized ( Humerickhouse et al., 2000; Slatter et al., 1997; Parvez et al., 2021; Satoh et al., 1994; Rivory et al., 1996 )
	CES2 $K_{M}$ (µM)	3.4	Humerickhouse et al. (2000)
	Biliary intrinsic clearance, ${\text{CL}}_{\mathrm{int},\text{bile}}$ (µL/min/ $10^{6}$ cells)	9	de Man et al. (2018), Tobin et al. (2005)
SN-38 (primary active metabolite).			
Physicochemical and blood binding	MW (g/mol)	392.4046	DrugBank
	log $P_{o:w}$	1.87	
	Compound type	Ampholyte	
	p $K_{a}$ 1	9.68	
	p $K_{a}$ 2	3.91	
	B/P ratio	0.6	Simcyp® prediction
	${\text{fu}}_{p}$	0.05	Chabot (1997)
Absorption (first-order model)	$P_{\text{eff},\text{man}}$ ( $10^{-4}$ cm/s)	0.63	Simcyp® prediction
	PSA (Å^2^)	99.96	DrugBank
	HBD	2	
Distribution (minimal PBPK model)	$V_{\text{SS}}$ (L/kg)	0.05	Optimized
Elimination – enzyme kinetics	UGTs (recombinant)		
	UGT1A1 $V_{\max}$ (pmol/min/mg protein)	212	Parvez et al. (2021), Xiao et al. (2018)
	UGT1A1 $K_{M}$ (µM)	2.6	Parvez et al. (2021), Xiao et al. (2018)
	UGT1A9 $V_{\max}$ (pmol/min/mg protein)	17	Parvez et al. (2021), Xiao et al. (2018)
	UGT1A9 $K_{M}$ (µM)	0.62	Parvez et al. (2021), Xiao et al. (2018)
SN-38G (secondary metabolite)			
Physicochemical and blood binding	MW (g/mol)	568.5	ADMET Predictor
	log $P_{o:w}$	−0.768	
	Compound type	Ampholyte	
	p $K_{a}$ 1	3.98	
	p $K_{a}$ 2	3.14	
	B/P ratio	0.66	
	${\text{fu}}_{p}$	0.75	
Distribution (minimal PBPK model)	$V_{\text{SS}}$ (L/kg)	0.09	Simcyp® prediction
	$K_{p}$ scalar	0.2	Optimized
Elimination – *in vivo* clearance	Hepatic clearance, ${\text{CL}}_{\text{iv}}$ (L/h)	2	Optimized
APC (primary inactive metabolite)			
Physicochemical and blood binding	MW (g/mol)	618	Santos et al. (2000)
	log $P_{o:w}$	2.108	ADMET Predictor
	Compound type	Ampholyte	
	p $K_{a}$ 1	3.81	
	p $K_{a}$ 2	9.91	
	B/P ratio	0.769	
	${\text{fu}}_{p}$	0.088	
Distribution (minimal PBPK model)	$V_{\text{SS}}$ (L/kg)	0.5	Optimized
Elimination – *in vivo* clearance	${\text{CL}}_{\text{iv}}$ (L/h)	20	Optimized

MW, molecular weight; B/P, blood-to-plasma; fu_p_, fraction unbound in plasma; P_eff,man_: Human jejunal permeability; PSA, polar surface area; HBD, hydrogen bond donors; V_SS_, volume of distribution at steady state; K_p_, tissue-to-plasma partition coefficient; CL_int_, intrinsic clearance; CL_int, bile_, intrinsic biliary clearance. Physicochemical and binding parameters were obtained from DrugBank, primary literature, Simcyp®, predictions, or ADMET, Predictor® as indicated. Optimized: parameter refined within predefined physiological bounds during model calibration.

Lopinavir/ritonavir combines UGT1A1 inhibition (lopinavir) with strong CYP3A inhibition (ritonavir) (Corona et al., 2008). In the within-cycle two-period clinical design, the observed pattern included a mild increase in CPT-11 and a larger increase in SN-38, effectively bridging the signatures of UGT1A1 and CYP3A inhibition. The model recovered this behavior within 2-fold for all CPT-11 and SN-38 endpoints, indicating that the dominant enzyme-mediated component of simultaneous conjugative and oxidative perturbation can be captured within the present framework, while acknowledging that transporter-related contributions to the net clinical interaction cannot be excluded. These interaction patterns are summarized in Table 4 under a dose-matched assumption, conceptualizing irinotecan clearance as parallel contributions from CES activation, CYP3A-mediated oxidation, and UGT1A1-mediated conjugation. Inhibition at the CYP3A node decreases APC formation and increases substrate availability for CES; inhibition at the UGT1A1 node directly reduces SN-38 clearance; and hypothetical inhibition of CES would be expected to increase CPT-11 exposure and decrease SN-38. Such directional signatures provide a qualitative framework for interpreting observed DDIs and anticipating the potential effects of future perpetrators acting on specific enzymatic nodes, while recognizing that non–dose-matched clinical designs may compress or invert apparent magnitudes even when the underlying mechanisms remain conserved.

**TABLE 4:** Exposure-signature matrix for the irinotecan under enzyme-specific perturbations (dose-matched assumption).

Mechanism/control node	Evidence level	CPT-11 _Cmax_	CPT-11 AUC	SN-38 _Cmax_	SN-38 AUC	References cases/Notes
UGT1A1 inhibition (clearance node)	Clinical	↑/≈	↑	↑↑	↑↑	SOR; LPV/RTV — SN-38 increases more than CPT-11 (see Table 3)
CYP3A inhibition (parallel branch)	Clinical	↑	↑	↑	↑	Ketoconazole — balanced increases; trial is non–dose-matched (350 mg/m^2^ alone vs. 100 mg/m^2^ + ketoconazole); interpret curves qualitatively, ratios per original design (Table 3)
CES inhibition (activation node)	No clinical DDI (hypothesis)	≈/↑	↑	↓	↓	No validated “pure CES inhibitor–CPT-11” study; list as theoretical only

↑ small–moderate increase; ↑↑ pronounced increase; ↓ decrease; ≈ no material change. Assumption: patterns reflect dose-matched comparisons. Non–dose-matched designs can compress or invert apparent magnitudes; interpret by direction and relative magnitude, not absolute levels. Clinical rows summarize net clinical interaction phenotypes interpreted within the enzyme-focused framework; transporter contribution cannot be excluded.

Two discrepancies emerged during model qualification. First, SN-38 AUC at 175 and 200 mg/m^2^ was overpredicted, with fold errors of 3.79 and 2.57, respectively. Because the overall dose–exposure regressions remained close to unity across the 33–750 mg/m^2^ range, these deviations are more likely attributable to study-specific factors or limitations in the representation of EHC and intestinal reactivation than to systematic bias in the activation or glucuronidation pathways. The EHC implementation preserves mass balance and the overall impact on systemic exposure but does not resolve segment-specific absorption, gut β-glucuronidase activity, or potential saturation within the EHC loop, all of which may disproportionately influence SN-38 at certain doses. Second, APC AUC under ketoconazole was markedly overpredicted (FE_AUC_ 3.811), despite adequate performance for CPT-11 and SN-38. APC physicochemical and clearance parameters relied primarily on in-silico estimates refined within physiological bounds during calibration. This discrepancy suggests remaining uncertainty in APC formation and disposition, and perhaps in the relative hepatic versus intestinal contributions to CYP3A-mediated metabolism under strong inhibition. Additional kinetic and distribution data for APC and related oxidative metabolites are needed to further constrain this pathway. Notably, APC is generally considered a pharmacologically inactive, minor metabolite of irinotecan, and its formation/disposition has been far less characterized than CPT-11 or SN-38 in the clinical and mechanistic literature. Accordingly, future work leveraging richer APC time-course or metabolite-profiling datasets (and, where appropriate, targeted CYP3A-pathway uncertainty/sensitivity assessments) would be most informative to better constrain this oxidative branch under strong inhibition.

Only a small fraction of an intravenous irinotecan dose (approximately 2%–5%) is converted to SN-38, reflecting the inherently low efficiency of activation (Chabot et al., 1995; Slatter et al., 1997). Under these conditions, hepatic and circulating CES1 contribute meaningfully to initial activation, despite CES2 having higher catalytic efficiency toward irinotecan and higher abundance in the intestine (Humerickhouse et al., 2000; Bencharit et al., 2002; Rivory et al., 1996). Plasma butyrylcholinesterase also exhibits activity toward CPT-11 (Rudakova et al., 2011), although its systemic contribution appears modest relative to hepatic CES. The present model captures these processes in aggregate using lumped CES-mediated intrinsic clearances scaled by tissue-specific enzyme abundances.

Clinically, dose-limiting gastrointestinal toxicity is correlated more strongly with intestinal SN-38 burden than systemic exposure alone (Di Martino et al., 2011; Chen et al., 2013; Teft et al., 2015). CES and UGTs enzymes are expressed in both liver and intestine (Rivory and Robert, 1995; Pommier, 2006; Ando et al., 2000; Hanioka et al., 2001; Jinno et al., 2003), transporters influence tissue distribution (Nakatomi et al., 2001; Lalloo et al., 2004; Nozawa et al., 2005; Fujita et al., 2016), and microbial β-glucuronidase can regenerate SN-38 from luminal SN-38G, amplifying local exposure (Bhatt et al., 2020). Enterohepatic cycling, with biliary excretion of CPT-11, SN-38, and SN-38G followed by intestinal deconjugation and reabsorption, is considered a major driver of this process (Takasuna et al., 1996; Wallace et al., 2010; Parvez et al., 2021).

In the present framework, however, EHC is implemented as an effective biliary intrinsic clearance term from liver to gut lumen without explicit modeling of gallbladder emptying, segment-specific absorption, or GUS-mediated deconjugation. This abstraction avoids overparameterization in the absence of robust in-vivo data but limits the model’s ability to capture regional intestinal SN-38 generation, secondary peaks, and saturation phenomena within the EHC loop. These omissions likely contribute to the residual SN-38 discrepancies at dose extremes and prevent mechanistic prediction of gastrointestinal toxicity. A dedicated intestinal/EHC module incorporating regional transport, metabolism, and microbial GUS activity would be required to address these limitations.

Several structural simplifications were implemented intentionally to maintain parameter identifiability and reduce the risk of over-fitting. First, transporters such as P-gp, BCRP, OATP1B1/1B3/2B1 were not represented as explicit mechanistic pathways. This decision was justified because in-vivo kinetic priors for these transporters in the irinotecan system are sparse and sometimes conflicting, and simultaneous estimation of multiple transporter modules alongside CES, UGT, and CYP pathways would be poorly identifiable and risk misleading mechanistic interpretations. This omission should not be interpreted as evidence that transporter effects are unimportant; rather, their relative contributions could not be uniquely resolved from the available plasma PK-based qualification datasets. Furthermore, EHC was not decomposed into explicit bile secretion, intestinal β-glucuronidase activity, or segment-specific reabsorption, leaving potential capacity limits or saturation within the EHC loop unrepresented. Such simplification may help preserve parsimony, but it may also contribute to underprediction of SN-38 at dose extremes and the inability to reproduce secondary peaks at the highest doses. In addition, APC parameters relied heavily on in-silico predictions refined within physiological limits, and the ketoconazole-associated discrepancy highlights the need for more comprehensive kinetic and distribution data for this metabolite. Finally, the model does not incorporate genotype stratification (e.g., UGT1A1*28 or *6), disease-related physiological alterations, or oncology-specific population characteristics such as reduced albumin, altered hepatic blood flow, or concomitant therapies. Accordingly, the observed clinical DDIs are interpreted here as net interaction phenotypes, for which enzyme perturbation is explicitly modeled and transporter contribution cannot be excluded. The current framework is therefore intended for enzyme-mediated DDI assessment under a nominally healthy physiological setting, rather than for transporter-dominant scenarios, comprehensive DDI prediction across all mechanisms, or formal exposure–toxicity modeling.

Future extensions may incorporate genotype-specific UGT1A1 activity, oncology-specific virtual populations, and a more mechanistic representation of EHC and intestinal processes. Such refinements would enable exploration of variability in SN-38 exposure across pharmacogenomic strata and disease states, as well as facilitate more direct linkage to gastrointestinal toxicity endpoints. Within its current scope, however, the model provides a transparent and quantitatively qualified platform for interpreting existing irinotecan DDI data and for conducting enzyme-network-informed “what-if” simulations to support regimen optimization and safety management.

## 5 Conclusion

This adult whole-body, middle-out PBPK model integrates CES-mediated activation, UGT1A1-driven glucuronidation, and CYP3A-mediated oxidation to describe systemic irinotecan disposition and its key metabolites. The model consistently reproduced plasma exposure across diverse monotherapy datasets and captured the direction and magnitude of enzyme-mediated interactions with ketoconazole, sorafenib, and lopinavir/ritonavir, aside from a persistent discrepancy for APC under strong CYP3A inhibition. Within its intended scope—predicting systemic exposure and enzyme-mediated drug-drug interactions rather than transporter-driven processes or gastrointestinal toxicity—the model provides a enzyme-focused transparent and quantitatively supported framework for interpreting irinotecan interaction data and performing exploratory dose-adjustment simulations, with future extensions incorporating genotype-specific UGT1A1 activity, oncology-tailored virtual populations, and mechanistic enterohepatic cycling expected to broaden its applicability.

## Data Availability

The original contributions presented in the study are included in the article/Supplementary Material, further inquiries can be directed to the corresponding authors.

## References

[B1] Abigerges D. Chabot G. G. Armand J. P. HéRAIT P. Gouyette A. Gandia D. (1995). Phase I and pharmacologic studies of the camptothecin analog Irinotecan administered every 3 weeks in cancer patients. J. Clin. Oncol. Official J. Am. Soc. Clin. Oncol. 13, 210–221. 10.1200/JCO.1995.13.1.210 7799022

[B2] Ando Y. Saka H. Ando M. Sawa T. Muro K. Ueoka H. (2000). Polymorphisms of UDP-Glucuronosyltransferase gene and Irinotecan toxicity: a pharmacogenetic analysis. Cancer Res. 60, 6921–6926. 11156391

[B3] Atasilp C. Chansriwong P. Sirachainan E. Reungwetwattana T. Puangpetch A. Prommas S. (2018). Determination of irinotecan, SN-38 and SN-38 glucuronide using HPLC/MS/MS: application in a clinical pharmacokinetic and personalized medicine in colorectal cancer patients. J. Clin. Lab. Anal. 32 (1), e22217. 10.1002/jcla.22217 28393405 PMC6817234

[B4] Bencharit S. Morton C. L. Howard-Williams E. L. Danks M. K. Potter P. M. Redinbo M. R. (2002). Structural insights into CPT-11 activation by Mammalian carboxylesterases. Nat. Struct. Biol. 9, 337–342. 10.1038/nsb790 11967565

[B5] Bhatt A. P. Pellock S. J. Biernat K. A. Walton W. G. Wallace B. D. Creekmore B. C. (2020). Targeted inhibition of gut bacterial β-glucuronidase activity enhances anticancer drug efficacy. Proc. Natl. Acad. Sci. U. S. A. 117, 7374–7381. 10.1073/pnas.1918095117 32170007 PMC7132129

[B6] Catimel G. Chabot G. G. Guastalla J. P. Dumortier A. Cote C. Engel C. (1995). Phase I and pharmacokinetic study of irinotecan (CPT-11) administered daily for three consecutive days every three weeks in patients with advanced solid tumors. Ann. Oncol. Official J. Eur. Soc. For Med. Oncol. 6, 133–140. 10.1093/oxfordjournals.annonc.a059108 7786821

[B7] Chabot G. G. (1997). Clinical pharmacokinetics of irinotecan. Clin. Pharmacokinet. 33, 245–259. 10.2165/00003088-199733040-00001 9342501

[B8] Chabot G. G. Abigerges D. Catimel G. Culine S. De Forni M. Extra J. M. (1995). Population pharmacokinetics and pharmacodynamics of irinotecan (CPT-11) and active metabolite SN-38 during phase I trials. Ann. Oncol. 6, 141–151. 10.1093/oxfordjournals.annonc.a059109 7786822

[B9] Chen S. Yueh M.-F. Bigo C. Barbier O. Wang K. Karin M. (2013). Intestinal glucuronidation protects against chemotherapy-induced toxicity by irinotecan (CPT-11). Proc. Natl. Acad. Sci. U. S. A. 110, 19143–19148. 10.1073/pnas.1319123110 24191041 PMC3839688

[B10] Corona G. Vaccher E. Sandron S. Sartor I. Tirelli U. Innocenti F. (2008). Lopinavir-ritonavir dramatically affects the pharmacokinetics of irinotecan in HIV patients with kaposi's sarcoma. Clin. Pharmacol. Ther. 83, 601–606. 10.1038/sj.clpt.6100330 17713471

[B11] De Jonge M. J. Verweij J. De Bruijn P. Brouwer E. Mathijssen R. H. Van Alphen R. J. (2000). Pharmacokinetic, metabolic, and pharmacodynamic profiles in a dose-escalating study of irinotecan and cisplatin. J. Clin. Oncol. Official J. Am. Soc. Clin. Oncol. 18, 195–203. 10.1200/JCO.2000.18.1.195 10623710

[B12] De Man F. M. Goey A. K. L. Van Schaik R. H. N. Mathijssen R. H. J. Bins S. (2018). Individualization of irinotecan treatment: a review of pharmacokinetics, pharmacodynamics, and pharmacogenetics. Clin. Pharmacokinet. 57, 1229–1254. 10.1007/s40262-018-0644-7 29520731 PMC6132501

[B13] Di Martino M. T. Arbitrio M. Leone E. Guzzi P. H. Rotundo M. S. Ciliberto D. (2011). Single nucleotide polymorphisms of ABCC5 and ABCG1 transporter genes correlate to irinotecan-associated gastrointestinal toxicity in colorectal cancer patients: a DMET microarray profiling study. Cancer Biol. and Ther. 12, 780–787. 10.4161/cbt.12.9.17781 21892003

[B14] Fan Y. Mansoor N. Ahmad T. Khan R. A. Czejka M. Sharib S. (2017). Physiologically based pharmacokinetic modeling for predicting irinotecan exposure in human body. Oncotarget 8, 48178–48185. 10.18632/oncotarget.18380 28636998 PMC5564636

[B15] Fan Y. Mansoor N. Ahmad T. Wu Z. X. Khan R. A. Czejka M. (2019). Enzyme and transporter kinetics for CPT-11 (irinotecan) and SN-38: an insight on tumor tissue compartment pharmacokinetics using PBPK. Recent Pat. Anti-cancer Drug Discov. 14, 177–186. 10.2174/1574892814666190212164356 30760193

[B16] Fujita D. Saito Y. Nakanishi T. Tamai I. (2016). Organic anion transporting polypeptide (OATP)2B1 contributes to gastrointestinal toxicity of anticancer drug SN-38, active metabolite of Irinotecan hydrochloride. Drug Metabolism Dispos. The Biol. Fate Chem. 44, 1–7. 10.1124/dmd.115.066712 26526067

[B17] Hanioka N. Ozawa S. Jinno H. Ando M. Saito Y. Sawada J. (2001). Human liver UDP-Glucuronosyltransferase isoforms involved in the glucuronidation of 7-ethyl-10-hydroxycamptothecin. Xenobiotica; Fate Foreign Compd. Biol. Syst. 31, 687–699. 10.1080/00498250110057341 11695848

[B18] Humerickhouse R. Lohrbach K. Li L. Bosron W. F. Dolan M. E. (2000). Characterization of CPT-11 hydrolysis by human liver carboxylesterase isoforms hCE-1 and hCE-2. Cancer Res. 60, 1189–1192. 10728672

[B19] Jinno H. Tanaka-Kagawa T. Hanioka N. Saeki M. Ishida S. Nishimura T. (2003). Glucuronidation of 7-ethyl-10-hydroxycamptothecin (SN-38), an active metabolite of irinotecan (CPT-11), by human UGT1A1 variants, G71R, P229Q, and Y486D. Drug Metabolism Dispos. The Biol. Fate Chem. 31, 108–113. 10.1124/dmd.31.1.108 12485959

[B20] Kehrer D. F. Mathijssen R. H. Verweij J. De Bruijn P. Sparreboom A. (2002). Modulation of irinotecan metabolism by ketoconazole. J. Clin. Oncol. 20, 3122–3129. 10.1200/JCO.2002.08.177 12118026

[B21] Lalloo A. K. Luo F. R. Guo A. Paranjpe P. V. Lee S.-H. Vyas V. (2004). Membrane transport of camptothecin: facilitation by human P-glycoprotein (ABCB1) and multidrug resistance protein 2 (ABCC2). BMC Med. 2, 16. 10.1186/1741-7015-2-16 15125776 PMC411064

[B22] Ma M. K. Mcleod H. L. (2003). Lessons learned from the irinotecan metabolic pathway. Curr. Med. Chem. 10, 41–49. 10.2174/0929867033368619 12570720

[B23] Mathijssen R. H. Van Alphen R. J. Verweij J. Loos W. J. Nooter K. Stoter G. (2001). Clinical pharmacokinetics and metabolism of irinotecan (CPT-11). Clin. Cancer Res. 7, 2182–2194. 11489791

[B24] Mathijssen R. H. Loos W. J. Verweij J. Sparreboom A. (2002). Pharmacology of topoisomerase I inhibitors irinotecan (CPT-11) and topotecan. Curr. Cancer Drug Targets 2, 103–123. 10.2174/1568009023333890 12188913

[B25] Minami H. Sai K. Saeki M. Saito Y. Ozawa S. Suzuki K. (2007). Irinotecan pharmacokinetics/pharmacodynamics and UGT1A genetic polymorphisms in Japanese: roles of UGT1A1*6 and *28. Pharmacogenet Genomics 17, 497–504. 10.1097/FPC.0b013e328014341f 17558305

[B26] Mross K. Steinbild S. Baas F. Gmehling D. Radtke M. Voliotis D. (2007). Results from an in vitro and a clinical/pharmacological phase I study with the combination irinotecan and sorafenib. Eur. J. Cancer 43, 55–63. 10.1016/j.ejca.2006.08.032 17095207

[B27] Nakatomi K. Yoshikawa M. Oka M. Ikegami Y. Hayasaka S. Sano K. (2001). Transport of 7-ethyl-10-hydroxycamptothecin (SN-38) by breast cancer resistance protein ABCG2 in human lung cancer cells. Biochem. Biophysical Res. Commun. 288, 827–832. 10.1006/bbrc.2001.5850 11688982

[B28] Nozawa T. Minami H. Sugiura S. Tsuji A. Tamai I. (2005). Role of organic anion transporter OATP1B1 (OATP-C) in hepatic uptake of Irinotecan and its active metabolite, 7-ethyl-10-hydroxycamptothecin: in vitro evidence and effect of single nucleotide polymorphisms. Drug Metabolism Dispos. The Biol. Fate Chem. 33, 434–439. 10.1124/dmd.104.001909 15608127

[B29] Parvez M. M. Basit A. Jariwala P. B. GáBORIK Z. Kis E. Heyward S. (2021). Quantitative investigation of Irinotecan metabolism, transport, and gut microbiome activation. Drug Metabolism Dispos. The Biol. Fate Chem. 49, 683–693. 10.1124/dmd.121.000476 PMC8407663 34074730

[B30] Pommier Y. (2006). Topoisomerase I inhibitors: camptothecins and beyond. Nat. Rev. Cancer 6, 789–802. 10.1038/nrc1977 16990856

[B31] Rivory L. P. (2000). Metabolism of CPT-11. Impact on activity. Ann. N. Y. Acad. Sci. 922, 205–215. 10.1111/j.1749-6632.2000.tb07039.x 11193896

[B32] Rivory L. P. Robert J. (1995). Molecular, cellular, and clinical aspects of the pharmacology of 20(S)camptothecin and its derivatives. Pharmacol. and Ther. 68, 269–296. 10.1016/0163-7258(95)02009-8 8719971

[B33] Rivory L. P. Bowles M. R. Robert J. Pond S. M. (1996). Conversion of irinotecan (CPT-11) to its active metabolite, 7-ethyl-10-hydroxycamptothecin (SN-38), by human liver carboxylesterase. Biochem. Pharmacol. 52, 1103–1111. 10.1016/0006-2952(96)00457-1 8831730

[B34] Rodgers T. Rowland M. (2007). Mechanistic approaches to volume of distribution predictions: understanding the processes. Pharm. Res. 24, 918–933. 10.1007/s11095-006-9210-3 17372687

[B35] Rudakova E. V. Boltneva N. P. Makhaeva G. F. (2011). Comparative analysis of esterase activities of human, mouse, and rat blood. Bull. Exp. Biol. Med. 152, 73–75. 10.1007/s10517-011-1457-y 22803044

[B36] Sai K. Kaniwa N. Ozawa S. Sawada J. I. (2001). A new metabolite of Irinotecan in which formation is mediated by human hepatic cytochrome P-450 3A4. Drug Metab. Dispos. 29, 1505–1513. 10.1124/dmd.29.11.1505 11602529

[B37] Santos A. Zanetta S. Cresteil T. Deroussent A. Pein F. Raymond E. (2000). Metabolism of irinotecan (CPT-11) by CYP3A4 and CYP3A5 in humans. Clin. Cancer Res. An Official J. Am. Assoc. For Cancer Res. 6, 2012–2020. 10815927

[B38] Satoh T. Hosokawa M. Atsumi R. Suzuki W. Hakusui H. Nagai E. (1994). Metabolic activation of CPT-11, 7-ethyl-10-[4-(1-piperidino)-1- piperidino]carbonyloxycamptothecin, a novel antitumor agent, by carboxylesterase. Biol. and Pharm. Bull. 17, 662–664. 10.1248/bpb.17.662 7920428

[B39] Shebley M. Sandhu P. Emami Riedmaier A. Jamei M. Narayanan R. Patel A. (2018). Physiologically based pharmacokinetic model qualification and reporting procedures for regulatory submissions: a consortium perspective. Clin. Pharmacol. Ther. 104, 88–110. 10.1002/cpt.1013 29315504 PMC6032820

[B40] Slatter J. G. Su P. Sams J. P. Schaaf L. J. Wienkers L. C. (1997). Bioactivation of the anticancer agent CPT-11 to SN-38 by human hepatic microsomal carboxylesterases and the in vitro assessment of potential drug interactions. Drug Metab. Dispos. 25, 1157–1164. 10.1124/dmd.25.10.1157 9321519

[B41] Sun R. Zhu L. Li L. Song W. Gong X. Qi X. (2020). Irinotecan-mediated diarrhea is mainly correlated with intestinal exposure to SN-38: critical role of gut ugt. Toxicol. Appl. Pharmacol. 398, 115032. 10.1016/j.taap.2020.115032 32387182 PMC9235850

[B42] Takasuna K. Hagiwara T. Hirohashi M. Kato M. Nomura M. Nagai E. (1996). Involvement of beta-glucuronidase in intestinal microflora in the intestinal toxicity of the antitumor camptothecin derivative Irinotecan hydrochloride (CPT-11) in rats. Cancer Res. 56, 3752–3757. 8706020

[B43] Tao G. Chityala P. K. Li L. Lin Z. Ghose R. (2022). Development of a physiologically based pharmacokinetic model to predict irinotecan disposition during inflammation. Chem. Biol. Interact. 360, 109946. 10.1016/j.cbi.2022.109946 35430260

[B44] Taskar K. S. Sharma P. Rowland Yeo K. (2025). Strategy for identifying rational sensitivity analysis using PBPK modeling for precipitant drug-drug interaction predictions. Clin. Pharmacol. Ther. 119 (2), 314–317. 10.1002/cpt.70127 41231089 PMC12816413

[B45] Teft W. A. Welch S. Lenehan J. Parfitt J. Choi Y. H. Winquist E. (2015). OATP1B1 and tumour OATP1B3 modulate exposure, toxicity, and survival after irinotecan-based chemotherapy. Br. J. Cancer 112, 857–865. 10.1038/bjc.2015.5 25611302 PMC4453959

[B46] Tobin P. J. Hong Y. Seale J. P. Rivory L. P. Mclachlan A. J. (2005). Loperamide inhibits the biliary excretion of irinotecan (CPT-11) in the rat isolated perfused liver. J. Pharm. Pharmacol. 57, 39–45. 10.1211/0022357055100 15638991

[B47] Toshimoto K. Tomaru A. Hosokawa M. Sugiyama Y. (2017). Virtual clinical studies to examine the probability distribution of the AUC at target tissues using physiologically-based pharmacokinetic modeling: application to analyses of the effect of genetic polymorphism of enzymes and transporters on Irinotecan induced side effects. Pharm. Res. 34, 1584–1600. 10.1007/s11095-017-2153-z 28397089 PMC5498655

[B48] Wallace B. D. Wang H. Lane K. T. Scott J. E. Orans J. Koo J. S. (2010). Alleviating cancer drug toxicity by inhibiting a bacterial enzyme. Science 330, 831–835. 10.1126/science.1191175 21051639 PMC3110694

[B49] Xiao L. Zhu L. Li W. Li C. Cao Y. Ge G. (2018). New insights into SN-38 glucuronidation: evidence for the important role of UDP glucuronosyltransferase 1A9. Basic Clin. Pharmacol. Toxicol. 122, 424–428. 10.1111/bcpt.12929 29076612

[B50] Xie R. Mathijssen R. H. J. Sparreboom A. Verweij J. Karlsson M. O. (2002). Clinical pharmacokinetics of irinotecan and its metabolites: a population analysis. J. Clin. Oncol. Official J. Am. Soc. Clin. Oncol. 20, 3293–3301. 10.1200/JCO.2002.11.073 12149304

[B51] Xu G. Zhang W. Ma M. K. Mcleod H. L. (2002). Human carboxylesterase 2 is commonly expressed in tumor tissue and is correlated with activation of irinotecan. Clin. Cancer Res. 8, 2605–2611. 12171891

