# Supplementary material for: Development of an adult whole-body PBPK model of irinotecan and its metabolites for predicting UGT1A1/CYP3A-mediated drug-drug interactions
Source: Front Pharmacol. 2026 May 26;17:1801897. doi: 10.3389/fphar.2026.1801897 (PMC13246723; doi:10.3389/fphar.2026.1801897)
Supplement: Supplementary file 1 [file Supplementaryfile1.docx]

# Supplementary Materials

**Figure S1** Model-predicted plasma concentration-time profiles of APC and SN-38G following 90-min IV irinotecan monotherapy at doses of 175-300 mg/m². Solid lines, median; dashed lines, 5th-95th percentiles.

**Figure S2** Model-predicted concentration–time profiles of APC and SN-38G under DDIs. Black: monotherapy; red: DDI.

**Table S1** PBPK Model Parameters for Ketoconazole

| **Category** | **Parameter** | **Value** |
| --- | --- | --- |
| **Physicochemical & Blood Binding** | Molecular weight (g/mol) | 531.4 |
|  | log $P_{o:w}$ | 4.04 |
|  | Compound type | Diprotic base |
|  | p$K_{a}$ 1 | 2.94 |
|  | p$K_{a}$ 2 | 6.51 |
|  | Blood-to-plasma ratio (B/P) | 0.62 |
|  | Fraction unbound in plasma ($\mathrm{fu}_{p}$) | 0.029 |
| **Absorption (First-order model)** | Fraction absorbed ($f_{a}$) | 1 |
|  | Absorption rate constant $k_{a}$ (1/h) | 0.78 |
|  | Enterocyte unbound fraction ($\mathrm{fu}_{\mathrm{Gut}}$) | 0.06 |
|  | $Q_{\mathrm{Gut}}$ (L/h) | 15.168 |
| **Distribution (Minimal PBPK)** | $V_{\mathrm{SS}}$ (L/kg) | 0.345 |
| **Elimination (In vivo clearance)** | Oral clearance $\mathrm{CL}_{\mathrm{po}}$ (L/h) | 14.4 |
|  | Active hepatic scalar (Net) | 2.07 |
|  | Renal clearance $\mathrm{CL}_{R}$ (L/h) | 0.147 |

**Table S2** PBPK Model Parameters for Sorafenib and Metabolite SNO

**Sorafenib**

| **Category** | **Parameter** | **Value** |
| --- | --- | --- |
| **Physicochemical & Blood Binding** | MW (g/mol) | 464.83 |
|  | log $P_{o:w}$ | 3.8 |
|  | Compound type | Monoprotic base |
|  | p$K_{a}$ 1 | 2.03 |
|  | B/P ratio | 0.75 |
|  | $\mathrm{fu}_{p}$ | 0.0023 |
| **Absorption (ADAM model)** | $P_{eff,man}$ (${10}^{-4}$ cm/s) | 1.5 |
| **Distribution (Full PBPK)** | $V_{\mathrm{SS}}$ (L/kg) | 0.772 |
| **Elimination (Enzyme kinetics)** | CYP3A4 $V_{\max}$ (pmol/min/pmol) | 18 |
|  | CYP3A4 $K_{M}$ (µM) | 6.1 |
|  | CYP3A4 ISEF | 0.55 |
|  | UGT1A9 $\mathrm{CL}_{\mathrm{int}}$ (µL/min/mg protein) | 5 |
|  | $\mathrm{CL}_{R}$ (L/h) | 0.003 |

**SNO (Sorafenib N-oxide)**

| **Category** | **Parameter** | **Value** |
| --- | --- | --- |
| **Physicochemical & Blood Binding** | MW (g/mol) | 480.83 |
|  | log $P_{o:w}$ | 2.165 |
|  | Compound type | Monoprotic base |
|  | p$K_{a}$ 1 | 2.03 |
|  | B/P ratio | 0.55 |
|  | $\mathrm{fu}_{p}$ | 0.006 |
| **Distribution (Minimal PBPK)** | $V_{\mathrm{SS}}$ (L/kg) | 0.124 |
| **Elimination (Enzyme kinetics)** | CYP3A4 $V_{\max}$ (pmol/min/pmol) | 1.72 |
|  | CYP3A4 $K_{M}$ (µM) | 11 |
|  | microsomal unbound fraction ($\mathrm{fu}_{\mathrm{mic}}$) | 0.35 |

**Table S3** PBPK Model Parameters for Ritonavir

| **Category** | **Parameter** | **Value** |
| --- | --- | --- |
| **Physicochemical & Blood Binding** | MW (g/mol) | 721 |
|  | log $P_{o:w}$ | 3.9 |
|  | Compound type | Diprotic base |
|  | p$K_{a}$ 1 | 1.8 |
|  | p$K_{a}$ 2 | 2.6 |
|  | B/P ratio | 0.587 |
|  | $\mathrm{fu}_{p}$ | 0.015 |
| **Absorption (First-order model)** | $f_{a}$ | 0.96 |
|  | $k_{a}$ (1/h) | 0.45 |
|  | $\mathrm{fu}_{\mathrm{Gut}}$ | 0.015 |
|  | $Q_{\mathrm{Gut}}$ (L/h) | 12.574 |
| **Distribution (Minimal PBPK)** | $V_{\mathrm{SS}}$ (L/kg) | 0.41 |
| **Elimination (In vivo clearance)** | CYP2D6 $V_{\max}$ (pmol/min/pmol) | 0.7 |
|  | CYP2D6 $K_{M}$ (µM) | 1 |
|  | CYP3A4 $V_{\max}$ (pmol/min/pmol) | 1.37 |
|  | CYP3A4 $K_{M}$ (µM) | 0.07 |
|  | CYP3A5 $V_{\max}$ (pmol/min/pmol) | 1 |
|  | CYP3A5 $K_{M}$ (µM) | 0.05 |
|  | HLM $\mathrm{CL}_{\mathrm{int}}$ (µL/min/mg protein) | 75 |
|  | $\mathrm{CL}_{R}$ (L/h) | 0.53 |

**Table S4** PBPK Model Parameters for Lopinavir

| **Category** | **Parameter** | **Value** |
| --- | --- | --- |
| **Physicochemical & Blood Binding** | MW (g/mol) | 628.1 |
|  | log $P_{o:w}$ | 4.2 |
|  | Compound type | Neutral |
|  | B/P ratio | 0.75 |
|  | $\mathrm{fu}_{p}$ | 0.01 |
| **Absorption (First-order model)** | $f_{a}$ | 1 |
|  | $k_{a}$ (1/h) | 30 |
|  | $\mathrm{fu}_{\mathrm{Gut}}$ | 1 |
|  | $Q_{\mathrm{Gut}}$ (L/h) | 15.015 |
| **Distribution (Minimal PBPK)** | $V_{\mathrm{SS}}$ (L/kg) | 0.82 |
| **Elimination (In vivo clearance)** | CYP3A4 $\mathrm{CL}_{\mathrm{int}}$ (µL/min/pmol) | 93.4 |
|  | $\mathrm{CL}_{R}$ (L/h) | 0.15 |

**Table S5** Parameterization of DDI mechanisms for each inhibitor model

| Enzymes | Inhibition | Parameters | Ketoconazole^a^ | SOR^b^ | SNO | RTV^c^ | LPV |
| --- | --- | --- | --- | --- | --- | --- | --- |
| CYP3A4 | Competitive | Ki (μM) | 0.015 | 0.015 | 15 | - | - |
|  |  | *fu_mic_* | 0.97 | 0.62 | 0.95 | - | - |
|  |  |  |  |  |  |  |  |
|  | Mechanism-Based | K_app_ (µM) | - | - | - | 0.18 | 0.41 |
|  |  | K_inact_ (1/h) | - | - | - | 19.8 | 1 |
|  |  |  |  |  |  |  |  |
| CYP3A5 | Mechanism-Based | K_app_ (µM) |  |  |  | 0.18 | 1 |
|  |  | K_inact_ (1/h) |  |  |  | 19.8 | 1 |
|  |  |  |  |  |  |  |  |
| UGT1A1 | Competitive | Ki (μM) | - | 0.027 | - | - | 0.03 |
|  |  |  |  |  |  |  |  |
| UGT1A9 | Competitive | Ki (μM) | - | 0.027 | - | - | - |

^a^：Reported Ki values in the literature span a relatively wide range, from 14.9 nM to 110 nM, depending on the substrate employed and the experimental system used.(Wrighton and Ring, 1994, Gibbs et al., 1999, Brown et al., 2007)

^b^：Previous studies have reported the PBPK parameters of sorafenib as a prototype inhibitor model(Wang et al., 2021), as well as its Ki against UGT1A(Miners et al., 2017).

^c^：Ritonavir (RTV) induces CYP3A4/5 (Ind_max_ = 68.5; Ind_C50_ = 1 μM). In the RTV/LPV regimen, CYP3A5 interaction parameters are retained because RTV is partially metabolized by CYP3A5 (auto-induction potential), and lopinavir (LPV) may inhibit RTV metabolism. The data were sourced from the Simcyp compound library.

**Table S6** Predicted versus observed Cmax and AUC for irinotecan (CPT-11) and SN-38 under monotherapy across all clinical studies.

| CPT-11 Dose (mg/m^2^) | n | Compound | Predicted Cmax (ng/mL) | | Predicted AUC (ng/mL·h) | | Observed Cmax (ng/mL) | | Observed AUC (ng/mL·h) | | FE_Cmax_ | FE_AUC_ | |
| --- | --- | --- | --- | --- | --- | --- | --- | --- | --- | --- | --- | --- | --- |
| 175 | 3 | CPT-11 |  | 2113.3 | | 11657.8 | | 2311.6 | | 10091.2 | 0.91 | | 1.16 |
|  |  | SN-38 |  | 46.3 | | 356.9 | | 29.4 | | 94.2 | 1.57 | | 3.79 |
|  |  | SN-38G |  | 239.9 | | 4012.3 | | 261.5 | | 4360.4 | 0.92 | | 0.92 |
|  |  | APC |  | 382.0 | | 4406.7 | | 290.8 | | 4009.2 | 1.31 | | 1.10 |
|  |  |  |  |  | |  | |  | |  |  | |  |
| 200 | 14 | CPT-11 |  | 2415.1 | | 13314.9 | | 2540.4 | | 12723.0 | 0.95 | | 1.05 |
|  |  | SN-38 |  | 52.0 | | 404.8 | | 35.3 | | 447.3 | 1.47 | | 0.90 |
|  |  | SN-38G |  | 270.2 | | 4550.2 | | 272.9 | | 4553.7 | 0.99 | | 1.00 |
|  |  | APC |  | 436.4 | | 5033.8 | | 321.7 | | 3650.3 | 1.36 | | 1.38 |
|  |  |  |  |  | |  | |  | |  |  | |  |
| 230 | 7 | CPT-11 |  | 2776.8 | | 15303.9 | | 3174.0 | | 15840.9 | 0.87 | | 0.97 |
|  |  | SN-38 |  | 58.5 | | 461.4 | | 35.3 | | 690.6 | 1.66 | | 0.67 |
|  |  | SN-38G |  | 305.5 | | 5186.5 | | 324.0 | | 4548.0 | 0.94 | | 1.14 |
|  |  | APC |  | 501.6 | | 5786.5 | | 556.8 | | 4231.9 | 0.90 | | 1.37 |
|  |  |  |  |  | |  | |  | |  |  | |  |
| 260 | 12 | CPT-11 |  | 3139.6 | | 17293.8 | | 2660.7 | | 13552.8 | 1.18 | | 1.28 |
|  |  | SN-38 |  | 64.7 | | 517.3 | | 43.2 | | 286.5 | 1.50 | | 1.81 |
|  |  | SN-38G |  | 339.7 | | 5813.1 | | 398.0 | | 4559.4 | 0.85 | | 1.27 |
|  |  | APC |  | 566.9 | | 6539.5 | | 451.7 | | 4559.8 | 1.26 | | 1.43 |
|  |  |  |  |  | |  | |  | |  |  | |  |
| 300 | 9 | CPT-11 |  | 3620.4 | | 19946.5 | | 4124.5 | | 21727.5 | 0.88 | | 0.92 |
|  |  | SN-38 |  | 72.6 | | 590.4 | | 51.0 | | 451.3 | 1.42 | | 1.31 |
|  |  | SN-38G |  | 383.7 | | 6633.9 | | 443.4 | | 7106.3 | 0.87 | | 0.93 |
|  |  | APC |  | 654.0 | | 7543.5 | | 513.5 | | 5902.4 | 1.27 | | 1.28 |
|  |  |  |  |  | |  | |  | |  |  | |  |
| 33 | 1 | CPT-11 |  | 785.2 | | 6702 | | 1919 | | 11418 | 0.41 | | 0.59 |
|  |  | SN-38 |  | 12.4 | | 213 | | 5.4 | | 171 | 2.29 | | 1.25 |
|  |  |  |  |  | |  | |  | |  |  | |  |
| 50 | 3 | CPT-11 |  | 1190 | | 10117 | | 1112 | | 11324 | 1.07 | | 0.89 |
|  |  | SN-38 |  | 18.3 | | 320 | | 10.9 | | 743 | 1.68 | | 0.43 |
|  |  |  |  |  | |  | |  | |  |  | |  |
| 66 | 9 | CPT-11 |  | 1567 | | 13333 | | 1186 | | 18604 | 1.32 | | 0.72 |
|  |  | SN-38 |  | 23.8 | | 419 | | 15.7 | | 725 | 1.51 | | 0.58 |
|  |  |  |  |  | |  | |  | |  |  | |  |
| 75 | 3 | CPT-11 |  | 1783 | | 15142 | | 1159 | | 13058 | 1.54 | | 1.16 |
|  |  | SN-38 |  | 26.7 | | 474 | | 19.6 | | 453 | 1.36 | | 1.05 |
|  |  |  |  |  | |  | |  | |  |  | |  |
| 85 | 4 | CPT-11 |  | 2021 | | 17152 | | 2494 | | 20962 | 0.81 | | 0.82 |
|  |  | SN-38 |  | 29.9 | | 535 | | 36.8 | | 628 | 0.81 | | 0.85 |
|  |  |  |  |  | |  | |  | |  |  | |  |
| 100 | 5 | CPT-11 |  | 2378 | | 20168 | | 2844 | | 28119 | 0.84 | | 0.72 |
|  |  | SN-38 |  | 34.6 | | 625 | | 42.9 | | 963 | 0.81 | | 0.65 |
|  |  |  |  |  | |  | |  | |  |  | |  |
| 115 | 4 | CPT-11 |  | 2732 | | 23185 | | 2034 | | 27857 | 1.34 | | 0.83 |
|  |  | SN-38 |  | 39.2 | | 715 | | 41.1 | | 843 | 0.95 | | 0.85 |
|  |  |  |  |  | |  | |  | |  |  | |  |
| 100 | 13 | CPT-11 |  | 2298 | | 6835 | | 2300 | | 5600 | 1.00 | | 1.22 |
|  |  | SN-38 |  | 33 | | 213 | | 34 | | 298 | 0.96 | | 0.72 |
|  |  |  |  |  | |  | |  | |  |  | |  |
| 150 | 4 | CPT-11 |  | 3443 | | 10218 | | 4100 | | 9200 | 0.84 | | 1.11 |
|  |  | SN-38 |  | 46 | | 314 | | 73 | | 514 | 0.64 | | 0.61 |
|  |  |  |  |  | |  | |  | |  |  | |  |
| 200 | 7 | CPT-11 |  | 4586 | | 13604 | | 2900 | | 9500 | 1.58 | | 1.43 |
|  |  | SN-38 |  | 59 | | 412 | | 32 | | 160 | 1.85 | | 2.57 |
|  |  |  |  |  | |  | |  | |  |  | |  |
| 230 | 8 | CPT-11 |  | 5276 | | 15637 | | 4600 | | 19700 | 1.15 | | 0.79 |
|  |  | SN-38 |  | 66 | | 469 | | 38 | | 264 | 1.74 | | 1.78 |
|  |  |  |  |  | |  | |  | |  |  | |  |
| 260 | 13 | CPT-11 |  | 5955 | | 17668 | | 4800 | | 22700 | 1.24 | | 0.78 |
|  |  | SN-38 |  | 73 | | 525 | | 112 | | 899 | 0.65 | | 0.58 |
|  |  |  |  |  | |  | |  | |  |  | |  |
| 300 | 5 | CPT-11 |  | 6870 | | 20378 | | 8700 | | 35300 | 0.79 | | 0.58 |
|  |  | SN-38 |  | 81 | | 599 | | 178 | | 966 | 0.46 | | 0.62 |
|  |  |  |  |  | |  | |  | |  |  | |  |
| 350 | 7 | CPT-11 |  | 8020 | | 23771 | | 7700 | | 34000 | 1.04 | | 0.70 |
|  |  | SN-38 |  | 91 | | 690 | | 56 | | 451 | 1.63 | | 1.53 |
|  |  |  |  |  | |  | |  | |  |  | |  |
| 400 | 7 | CPT-11 |  | 9172 | | 27162 | | 9300 | | 42900 | 0.99 | | 0.63 |
|  |  | SN-38 |  | 100 | | 778 | | 94 | | 665 | 1.07 | | 1.17 |
|  |  |  |  |  | |  | |  | |  |  | |  |
| 450 | 4 | CPT-11 |  | 10316 | | 30556 | | 17300 | | 37900 | 0.60 | | 0.81 |
|  |  | SN-38 |  | 109 | | 865 | | 299 | | 2214 | 0.36 | | 0.39 |
|  |  |  |  |  | |  | |  | |  |  | |  |
| 500 | 10 | CPT-11 |  | 11456 | | 33951 | | 11700 | | 42900 | 0.98 | | 0.79 |
|  |  | SN-38 |  | 117 | | 950 | | 226 | | 1159 | 0.52 | | 0.82 |
|  |  |  |  |  | |  | |  | |  |  | |  |
| 600 | 9 | CPT-11 |  | 13739 | | 40744 | | 11200 | | 53000 | 1.23 | | 0.77 |
|  |  | SN-38 |  | 132 | | 1115 | | 174 | | 1821 | 0.76 | | 0.61 |
|  |  |  |  |  | |  | |  | |  |  | |  |
| 750 | 7 | CPT-11 |  | 17215 | | 50947 | | 13000 | | 67700 | 1.32 | | 0.75 |
|  |  | SN-38 |  | 152 | | 1351 | | 149 | | 2370 | 1.02 | | 0.57 |
|  |  |  |  |  | |  | |  | |  |  | |  |

# REFERENCES

BROWN, H. S., CHADWICK, A. & HOUSTON, J. B. 2007. Use of isolated hepatocyte preparations for cytochrome P450 inhibition studies: comparison with microsomes for Ki determination. *Drug Metab Dispos,* 35**,** 2119-26.

GIBBS, M. A., THUMMEL, K. E., SHEN, D. D. & KUNZE, K. L. 1999. Inhibition of cytochrome P-450 3A (CYP3A) in human intestinal and liver microsomes: comparison of Ki values and impact of CYP3A5 expression. *Drug Metab Dispos,* 27**,** 180-7.

MINERS, J. O., CHAU, N., ROWLAND, A., BURNS, K., MCKINNON, R. A., MACKENZIE, P. I., TUCKER, G. T., KNIGHTS, K. M. & KICHENADASSE, G. 2017. Inhibition of human UDP-glucuronosyltransferase enzymes by lapatinib, pazopanib, regorafenib and sorafenib: Implications for hyperbilirubinemia. *Biochemical Pharmacology,* 129**,** 85-95.

WANG, Z., XIANG, X., LIU, S., TANG, Z., SUN, H., PARVEZ, M., GHIM, J. L., SHIN, J. G. & CAI, W. 2021. A physiologically based pharmacokinetic/pharmacodynamic modeling approach for drug-drug interaction evaluation of warfarin enantiomers with sorafenib. *Drug Metab Pharmacokinet,* 39**,** 100362.

WRIGHTON, S. A. & RING, B. J. 1994. Inhibition of human CYP3A catalyzed 1'-hydroxy midazolam formation by ketoconazole, nifedipine, erythromycin, cimetidine, and nizatidine. *Pharm Res,* 11**,** 921-4.
